# Faculty, staff, and student perceptions of substance use disorder stigma in health profession training programs: a quantitative study

**DOI:** 10.1186/s13011-022-00509-8

**Published:** 2023-01-06

**Authors:** Rachel E. Barenie, Alina Cernasev, R. Eric Heidel, Steven Stewart, Kenneth Hohmeier

**Affiliations:** 1 College of Pharmacy, University of Tennessee Health Science Center, Memphis, TN USA; 2 Department of Surgery, Office of Medical Education, Research, and Development, University of Tennessee Graduate School of Medicine, Knoxville, TN USA; 3 Big Tree Medical, Columbia, MO USA

**Keywords:** Stigma, Substance use disorder, Health profession education

## Abstract

**Background:**

Research indicates that stigma impacts the care provided to individuals with Substance Use Disorders (SUDs), but perceptions of SUDs in various healthcare training programs are not well known. We aimed to characterize perceptions of faculty, staff, and students about SUD stigma in professional healthcare training programs.

**Methods:**

We conducted a cross-sectional survey of faculty, staff, and students employed at or enrolled in one of six health-related colleges at one Mid-South health science center in the United States, including medicine, pharmacy, dentistry, nursing, health professions, and graduate health sciences. Data collection occurred between February and March 2021. We used descriptive and frequency statistics to assess the constructs within the survey instrument.

**Results:**

A total of 572 respondents participated in this study (response rate = 9%; students, *n* = 428, 75%; faculty, *n* = 107, 19%; staff, *n* = 32, 6%). Most respondents reported interacting with persons with a SUD, cited challenges with the interaction, and perceived SUDs to be mental health condition (*n* = 463) or biological disease (*n* = 326). Most respondents believed that their college: emphasizes learning about SUDs; promotes an accurate perception of SUDs; and fosters respect for persons with. Few respondents reported they hear faculty, staff, or students express negative comments about persons with SUDs, but they were sometimes expressed by students.

**Conclusions:**

Most faculty, staff, and students reported experiencing challenges when interacting with a person with a SUD, mainly communication, but few recalled hearing negative comments from their peers. Whether interventions tailored towards improving communication in academic healthcare training settings could minimize challenges experience by faculty, staff, and students when serving individuals with SUDs should be further evaluated.

## Background

Substance use disorders (SUDs) are some of the most stigmatized conditions in the world [1]. Stigma occurs when a person is labeled, stereotyped, or discriminated against, and it is one of the leading predictors of poor health outcomes for people with SUDs [2, 3]. For example, SUD-related stigma may decrease a person’s willingness to seek treatment because of the fear or shame they report experiencing [4, 5]. Even when a person seeks treatment, the access to, type of, and perceptions about that treatment may vary based on their race, gender, sexual orientation and other factors [6–8].

Stigma does not only affect the person with a SUD, but it can also negatively influence a healthcare practitioner’s beliefs. This in turn may impact the way a healthcare practitioner cares for their patients, such as how they engage with a patient or select the best treatment option, and even impact patient outcomes [9]. How student healthcare practitioners form their views on SUDs is complex and multifactorial. Studies have shown that negative attitudes toward individuals with SUDs tend to worsen during residency training, such as in psychiatry [10, 11]. Thus, there may be an opportunity during the formative years of a healthcare professional training program to influence practitioner perceptions about SUDs. For instance, language choice directly reflects how a person perceives an issue, and the literature demonstrates that language used to discuss SUDs—with patients, colleagues, and among peers—is key to combat the stigma associated with the disease [12–14]. Recent data also suggests that additional healthcare provider training on SUDs could actually reverse that trend and improve provider attitudes towards people with SUDs [15].

Research suggests support staff perceptions about people with SUDs may also influence the care patients with SUDs receive in practice [16, 17]. Considering support staff are integral to the patient care process, this phenomenon may extend to institutes of higher education, and it signals that a “hidden curriculum” may exist that perpetuates the stigma associated with SUDs in professional healthcare training programs [18]. Since institutes of higher education serve as the starting point for the majority of future healthcare practitioners, researchers have posited that trainees may be “agents of change” to address the opioid epidemic as well as address other barriers to evidence-based care [19]. Furthermore, this positive change could be led by the faculty and staff at these educational programs where students learn, considering all three—faculty, staff, and students—influence the planned and hidden curriculums at their institution.

While much research exists linking stigma to lower quality care for people with SUDs, perceptions of faculty, staff, and students in professional healthcare training programs is not well known. Thus, we aimed to characterize their perceptions at one Mid-South health science center in the United States.

## Methods

We conducted a cross-sectional, self-administered survey of faculty, staff, and students at six health-related colleges at one Mid-South health science center in the United States, including medicine, pharmacy, dentistry, nursing, health professions, and graduate health sciences. Faculty included respondents with a faculty appointment of Instructor, Assistant Professor, Associate Professor, or Professor. Staff included respondents without a faculty appointment that served in an administrative role. Finally, students included respondents that were enrolled in one of the six health science colleges at the time the study was conducted. Data collection occurred between February and March 2021. The University of Tennessee Institutional Review Board (IRB # 21–07,977-XM, March 1^st^ 2021) granted approval for this study. The survey responses were captured and stored electronically. At the beginning of the survey, the respondents read and consented to participate into the study. The survey respondents could choose not to respond to any questions within the survey. The survey was distributed over 2021. The survey invitation was sent two times within four weeks in between the initial email.

### Survey instrument development

The survey consisted of several parts and was composed of 50-questions, including Likert scale, multiple-choice question, closed questions (Appendix 1) [20]. The survey instrument centered on the following domains based on concepts from the Stigma Conceptualization framework proposed by Link and Phelan: attitudes, behaviors, perceptions, and stigma towards SUDs [21]. The instrument did not contain any open-ended questions and finished with demographic items. We modified the wording of the questions to resonate with the healthcare environment. from previously published surveys [22–26]. The survey was peer-reviewed by a SUD working group of pharmacists and non-pharmacist practitioners, with small adjustments made for question clarity based on their feedback before it was administered.

The survey included questions about the following: participant’s role at the college (faculty, staff, or student)(1 question total; multiple choice); perceptions of SUDs (9 questions total; 7 multiple choice; 2 Likert-scale questions); perceptions of stigma in health professional training programs (8 questions total; 8 Likert-scale questions [1 = strongly agree to 5 = strongly disagree]); stigma experiences at the institution (8 questions total; 8 Likert-scale questions [5 were 1 = strongly agree to 5 = strongly disagree; 3 were 1 = never to 5 = always]); existing efforts to thwart stigma at the institution (4 questions total; 4 Likert-scale questions); and participant characteristics (15 questions total; 12 multiple choice questions; 3 open-ended questions) (Appendix 1).

### Recruitment

Faculty, staff, and students were recruited from all six health profession Colleges at one Mid-South health science center in the United States. We initially recruited an individual at each College to help facilitate efficient dissemination of the survey via email and improve credibility, commonly an administrative leader. The survey was shared with the individual identified to distribute to their audience via email three times. Each recruitment email was separated by a two-week period [20]. We also invited participants through the institution’s campus-wide email system that is sent to faculty, staff, and students. Of those participants who completed the survey, we randomly selected one participant to win a $250 gift card and fifteen other participants to win a $50 gift card.

### Data analysis

Frequency and percentage statistics were used to analyzed the categorical variables in the survey instrument. Descriptive statistics were performed for continuous parameters, mainly means and standard deviations. Kruskal–Wallis tests were performed to compare independent groups on survey item values. When a significant main effect was detected, post hoc testing was performed using Dunn’s test. Medians and interquartile ranges were reported and interpreted for the non-parametric analyses. All statistical analyses were performed using SPSS Version 28 (Armonk, NY: IBM Corp.).

## Results

A total of 572 respondents participated in this study (overall response rate = 9%). Most respondents were students (*n* = 428; 75%), followed by faculty (*n* = 107; 19%) and staff (*n* = 32; 6%) (Table 1). Most respondents were female (*n* = 372; 76%), the highest degree completed was a 4-year college degree (*n* = 308; 52%), 31 years old on average (Standard Deviation [SD]: 12.2 years), not Hispanic, Latino or Spanish origin (*n* = 473; 96%), and white (*n* = 336; 69%) (Table 1). Most respondents were from the college of pharmacy (*n* = 214; 43%), followed by medicine (*n* = 101; 21%), nursing (*n* = 74; 15%), health professions (*n* = 50; 10%), dentistry (*n* = 29; 6%), and graduate health sciences (*n* = 24; 5%) (Table 1).

**Table 1:** Respondent Characteristics

Characteristics	n	%
Role		
Faculty	107	19
Staff	32	6
Student	428	75
Age (years)	31 (SD: 12)	
Sex		
Male	114	23
Female	372	76
Prefer not to answer	6	1
Hispanic, Latino, or Spanish Origin		
Yes	19	4
No	473	96
Race		
White	336	69
Black or African American	75	15
Asian Indian	27	6
Some other race*	51	10
College		
Dentistry	29	6
Graduate Health Sciences	24	55
Health Professions	50	10
Medicine	101	21
Nursing	74	15
Pharmacy	214	43

^*^Race categories with a frequency of 15 respondents or less were consolidated to the “Some other race” category

### SUD perceptions

Most respondents reported knowing someone with a SUD (*n* = 340; 60%), and, of those, nearly all participants had interacted with someone with a SUD (*n* = 333; 99%) and did so on a weekly basis (*n* = 126; 38%), followed by monthly (*n* = 98; 30%), quarterly (*n* = 55; 17%), annually (*n* = 46; 14%), or never (*n* = 4; 1%). Those interactions most commonly occurred in a personal setting (*n* = 311; 53%), followed by a professional setting (*n* = 188; 32%) or another type of setting, such as in the general public or community service events (*n* = 6; 1%). Of respondents who reported interacting with a person with a SUD, many reported experiencing challenges interacting with that person (*n* = 276; 48%). The most common cited challenge was difficulty communicating (*n* = 211; 37%), followed by lack of trust on the part of the individual (*n* = 186; 33%), overall uncomfortability (*n* = 110; 19%), inadequate amount of time to spend with person (*n* = 80; 14%), overall feeling of being unprepared (*n* = 59; 10%), or another reason (*n* = 23; 4%).

When asked about their perceptions of SUDs, most respondents answered that they viewed a SUD as a mental health condition (*n* = 463; 81%), followed by a biological disease (*n* = 326; 57%), a consequence of the family or community where one lives (*n* = 328; 57%), a personal choice (*n* = 214; 37%), a moral failing (*n* = 42; 7%), or were not sure (*n* = 39; 7%) (Table 2). Staff more commonly than faculty or students perceived SUDs as a moral failing or personal choice whereas faculty and students more commonly perceived it as a biological disease or mental health condition (Table 2). Most respondents felt very or somewhat comfortable continuing a friendship with someone in their college that has a SUD (*n* = 327; 60%), with faculty significantly more likely than staff to feel comfortable continuing the relationship, H(2) = 16.82, *p* < 0.001 (main effect), post hoc Dunn’s test, z = -4.10, *p* < 0.001. Few respondents reported being very likely or likely to avoid a colleague that has a SUD (*n* = 66; 12%), with students significantly more likely than faculty to avoid a colleague, H(2) = 13.52, *p* = 0.001 (main effect), post hoc Dunn’s test, z = 3.60, *p* < 0.001.

**Table 2:** Faculty, Staff, and Student Perceptions of SUD.*

	Perception of SUD									
	A moral failing		A personal choice		A biological disease		A consequence of the family of community where one lives		A mental health condition	
Role	n	%	n	%	n	%	n	%	n	%
Faculty	4	4	20	19	63	59	45	42	73	68
Staff	4	13	12	38	9	28	13	41	20	63
Student	29	7	155	36	218	51	232	54	321	75

^*^Respondents were permitted to select more than one answer option

### Perceptions of stigma in health professional training

Respondents were also asked about their perceptions of stigma in health professional training. Most respondents strongly agreed, agreed, or somewhat agreed that they felt comfortable engaging with someone with a SUD (*n* = 505; 94%). When asked if they perceive people with SUDs similar to a people with other disease states, such as high blood pressure, most strongly agreed, agreed, or somewhat agreed that they did (*n* = 425; 79%), and only a few disagreed (*n* = 97; 18%) or strongly disagreed (*n* = 13; 2%). Faculty were significantly more likely than students to perceive people with SUDs similarly to people with other disease states, H(2) = 8.37, *p* = 0.015 (main effect), post hoc Dunn’s test, z = -2.85, *p* = 0.004.

Most respondents agreed that they believe their college emphasizes learning about SUDs (strongly agree, *n* = 98, 18%; agree, *n* = 175, 33%; somewhat agree, *n* = 140, 26%). Most respondents also agree that their college promotes an accurate perception of SUDs (strongly agree, *n* = 85, 16%; agree, *n* = 206, 39%; somewhat agree, *n* = 163, 31%). Also, most respondents reported that they believe their college fosters respect for people with SUDs (strongly agree, *n* = 101, 19%; agree, *n* = 209, 39%; somewhat agree, *n* = 155, 29%).

Faculty, staff, and students were also probed about whether they believe people in those roles at their institution hold negative perceptions of people with SUDs. Most respondents agreed that they believe most faculty at their college do not hold negative perceptions of people with SUDs (strongly agree, *n* = 75, 14%; agree, *n* = 192, 36%; somewhat agree, *n* = 173, 33%; disagree, *n* = 81, 15%; strongly disagree, *n* = 11, 2%), H(2) = 21.34, *p* < 0.001, with students significantly more likely than faculty (z = 4.03, *p* < 0.001) and students significantly more likely than staff (z = 2.70, *p* = 0.007) to believe this. Most respondents agreed that they believe most staff at their college do not hold negative perceptions of people with SUDs (strongly agree, *n* = 59, 11%; agree, *n* = 198, 37%; somewhat agree, *n* = 186, 35%; disagree, *n* = 75, 14%; strongly disagree, *n* = 14, 2%), H(2) = 22.05, *p* < 0.001. Post hoc testing with Dunn’s test showed students were significantly more likely than staff (z = 2.01, *p* = 0.045) and, separately, faculty (z = 4.45, *p* < 0.001) to believe this. Most respondents agreed that they believe most staff at their college do not hold negative perceptions of people with SUDs (strongly agree, *n* = 30, 6%; agree, *n* = 163, 31%; somewhat agree, *n* = 210, 39%; disagree, *n* = 114, 21%; strongly disagree, *n* = 16, 3%). No findings were significant.

Most respondents overall reported that they never or rarely heard faculty, staff, or students express negative comments about people with SUDs (faculty, never = 345, 67%; rarely = 121, 23%; staff, never = 353, 68%, rarely = 112, 22%; students, never = 211, 41%, rarely = 167, 32%). Some respondents reported that they sometimes (*n* = 118, 23%) or often heard students express negative comments (*n* = 21, 4%). Very few respondents report hearing faculty or staff express negative comments sometimes or often (faculty, sometimes = 45, 9%; often = 6,1%; staff, sometimes = 44, 9%; often = 7, 1%).

### Stigma experiences at my own institution

Respondents were prompted to reflect on how hearing negative comments would make them feel, and most agreed that it would make them feel uncomfortable (*n* = 455; 88%). Most respondents also agreed to some degree that they have felt uncomfortable about the negative comments they have heard at their College about people with SUDs (strongly agreed, *n* = 39, 8%; agreed, *n* = 177, 34%; somewhat agreed *n* = 122, 24%). Most respondents reporting feeling confident in thinking and expressing their own opinions about people with SUDs (*n* = 475; 92%) and feel empowered to take action to address comments that may make them feel uncomfortable about people with SUDs (*n* = 439; 85%). Most even agreed that they have taken action to address comments that made them feel uncomfortable (strongly agree, *n* = 52, 10%; agree, *n* = 141, 27%; somewhat agree, *n* = 144, 28%; disagree, *n* = 153, 26%; strongly disagree, *n* = 26, 4%). When respondents were asked to reflect on whether they heard faculty, staff, or students express negative comments about people with SUDs, most reported they never or rarely heard negative comments from all three groups.

### Existing efforts to thwart stigma

Respondents were also queried about the levels of emotional distress they experienced related to the stigma they perceived on at their institution. Few participants reported experiencing any emotional distress at all related to stigma perceived or experienced related to SUDs (never: *n* = 248, 50%; rarely: *n* = 159, 32%; sometimes: *n* = 74, 15%; often: *n* = 15, 3%; always: *n* = 1; 0.2%). Most respondents reported that the emotional did not interfere with their daily activities (not at all: *n* = 372, 75%; a little: *n* = 86, 17%; moderately: *n* = 30, 6%; quite a bit: *n* = 8, 1.6%; extremely: *n* = 1, 0.2%).

## Discussion

This study provides important insights on views of SUD in health professions training environments—especially in providing a baseline of faculty, staff, and student perceptions as well as potential targets for future training or education. Most respondents reported perceiving a SUD as mental health condition, and many respondents also reported experiencing communication challenges when interacting with patients with SUDs. Most respondents also reported that they do not hear faculty, staff, or students express negative comments about people with SUDs, but a few did. Whether interventions tailored at improving communication in the academic healthcare training settings could minimize challenges when serving people with SUDs should be further evaluated.

Our questionnaire probed for the first time how faculty, staff, and students perceive SUDs, and our findings highlight that most faculty, staff, and students view a person with SUD similar to a person with other disease states, such as high blood pressure. Even though a SUD is an established disease with nationally accepted diagnostic criteria defined in The Diagnostic and Statistical Manual of Mental Disorders, Fifth Edition (DSM-5), there were still one in five respondents viewed these people differently [27]. A previous study measured the stigma toward opioid use and reported that young people believe that a moderate amount of discrimination toward individuals with opioid use disorder is acceptable. The study also found a negative link between stigma and family misuse of opioids [28]. This is important because the way individuals perceive a problem influences the language they use address it, which may later inform their approach to action or care provided. Furthermore, a survey by Link et al. reported the importance of understanding the stigma mechanisms and how they affect people with whom they interact [29]. Although its implications are uncertain, one salient finding was that staff were twice as likely to find SUD as a moral failing as either students or faculty. Despite not having a direct role in student training or patient care, staff may influence both faculty and student perceptions as a key stakeholder in the learning environment, and this is worth further exploration. Thus, training every member of the educational team—faculty, staff, and students—may be key to eliminate the detrimental perceptions of people with SUDs that may exist. Tailored interventions for the academic healthcare training setting should be explored to address the perceptions held by some members of the educational team.

Additionally, previous research has demonstrated that how individuals perceive people with SUDs can, in fact, change over time [30]. Most respondents reported experiencing challenges when interacting with a person with a SUD, with communication being the most frequently cited challenge by all respondents. Implementing tailored interventions, such as specific training on the topic of communicating with people with SUDs, should be explored. One prior study assessed resident attitudes before and after an online training program about stigma [4]. The researchers measured the residents attitudes using the Medical Condition Regard Scale (MCRS), and found that the resident’s attitudes six months after the training had improved [4]. Considering approximately 14.5% of the US population ages 12 years or older had an SUD in 2020, the likelihood of encountering a person with a SUD is high [31]. This not only reaffirms the need for targeted training for all members of the educational team, but it highlights the positive and lasting impact such training could have over time.

The potential impact that tailored communication training could have on individual behavior is clear, and our research highlights a need for such training remains. Components of such a training intervention should be tailored to their specific audience, taking into account their role on the healthcare team. One foundational component includes specific training on how the verbal communication, such as the words they use to describe something as simple as the results of urine drug test, is a direct reflection of how an individual perceives an issue. There are many resources available to support training on verbal communication when discussing the disease of addiction [32], and some researchers have even proposed an “addiction-ary” [12]. Since professional training programs will ultimately graduate the future generations of healthcare professionals, proactively ensuring appropriate training focused on communication for every member of the educational team is a necessary component of curriculums—both planned and hidden.

### Limitations

This study has several limitations. First, all survey respondents were employed or enrolled in one Mid-South health science center in the United States, which limits the generalizability of our results. Second, due to our recruitment methods (identifying champions at each college to disseminate the invitation to participate and posting it to the daily digest emailed to all), we do not know how exactly how individuals in total received the invitation email. Thus, we used aggregate data from the institution for the total number of faculty, staff, and students to calculate the response rate. Third, our recruitment time period did overlap with the institutions planned Spring Break, which may have limited its uptake. Also, survey fatigue due to COVID-19 may have also impacted the response rate, which has been demonstrated by prior research. Fourth, our survey did not collect information about training respondents already received on this topic. Fifth, non-response bias may have influenced the study’s findings, as faculty, staff and/or students at the health science center who did not respond to the survey may have answered questions differently—this is especially true of faculty and staff response rates which were both under 20%.

## Conclusions

Most faculty, staff, and students reported perceiving a SUD as a mental health condition, but commonly reported experiencing challenges when interacting with people with SUDs, frequently noting communication. Whether interventions tailored at improving communication skills in academic healthcare training settings could minimize challenges when serving people with SUDs should be further evaluated.

## Data Availability

Not applicable.

## Appendix 1. Survey distributed to the participants

Participant Role at UTHSC.

1. What is your role at UTHSC?
  - Faculty
  - Staff
  - Student
  - Other (please specify: _______________)

Substance Use Disorder Perceptions.

1. Do you know someone that has a substance use disorder?
  - Yes
  - No
    If yes, then proceed to question 2.
    If no, then skip to question 7.
2. Have you interacted with someone that has a substance use disorder?
  - Yes
  - No
    If yes, then proceed to question 3.
    If no, then skip to question 7.
3. In what setting have you interacted with a person with a substance use disorder?
  - Professional
  - Personal
  - Other (please specify: __________________)
4. How frequently do you interact with persons with a substance use disorder?
  - Weekly
  - Monthly
  - Quarterly
  - Annually
  - Never
5. Have you experienced challenges interacting with a person that has a substance use disorder?
  - Yes
  - No
    If yes, then proceed to question 6.
    If no, then skip to question 7.
6. What challenges did you experience? (select all that apply)
  - Difficulty communicating
  - Lack of trust on the part of the individual
  - Overall uncomfortable
  - Overall unprepared
  - Inadequate amount of time to spend with person
  - Other (Please specify: ______________________)
7. How do you perceive substance use disorders?
  - A moral failing
  - A personal choice
  - A biological disease
  - A consequence of the family or community where one lives
  - A mental health condition
  - I am not sure.
  - Other (please specify: ____________________)
8. What is your comfort level in continuing a friendship if you found out someone in your College has a substance use disorder?
  - Very comfortable
  - Somewhat comfortable
  - Neutral
  - Somewhat Uncomfortable
  - Very uncomfortable
9. How likely would you be to avoid your colleague if you found out that he/she had a substance use disorder?
  - Very likely
  - Likely
  - Neutral
  - Unlikely
  - Very unlikely

Reflecting on your experiences at UTHSC in the last 3 months, please indicate the extent to which you agree or disagree. Answers: (1) Strongly agree, (2) Disagree, (3) Somewhat agree, (4) Agree, (5) Strongly disagree.

1. I feel comfortable engaging with someone with a substance use disorder.
2. I perceive persons with substance use disorders similarly to persons with other disease states, like high blood pressure.
3. I believe my College emphasizes learning about substance use disorders in the curriculum.
4. I believe my College promotes an accurate perception of substance use disorders.
5. I believe my College fosters respect for persons with substance use disorders.
6. I believe most faculty at my College do not have negative perceptions of persons with substance use disorders.
7. I believe most staff at my College do not have negative perceptions of persons with substance use disorders.
8. I believe most students at my College do not have negative perceptions of persons with substance use disorders.

Stigma Experiences at UTHSC.

For the following questions, please reflect on your experiences at UTHSC in the last 3 months.

Please indicate the extent to which you agree or disagree. Answers: (1) Strongly agree, (2) Disagree, (3) Somewhat agree, (4) Agree, (5) Strongly disagree.

1. If I hear anyone express a negative comment about a person with a substance use disorder, then I would feel uncomfortable.
2. I feel uncomfortable about negative comments that I have heard at my College about persons with substance use disorders.
3. I feel confident to think and express my own opinions about persons with substance use disorders.
4. I feel empowered to take action to address comments that may make me feel uncomfortable about persons with substance use disorders.
5. I have taken action to address comments that did make me feel uncomfortable about persons with substance use disorders.

Suppose you are on the UTHSC campus, please indicate how often you recall the following experiences. Answers: (1) Never, (2) Rarely, (3) Sometimes, (4) Often, (5) Always.

1. I hear faculty express negative comments about persons with a substance use disorder.
2. I hear staff express negative comments about persons with a substance use disorder.
3. I hear students express negative comments about persons with a substance use disorder.

Existing Efforts to Thwart Stigma.

1. In the past 3 months, how often have you felt emotional distress related to stigma you perceived or experienced regarding substance use disorders.
  - Never
  - Rarely
  - Sometimes
  - Often
  - Always
2. In the past 3 months, how much did the distress interfere with your daily activities?
  - Not at all
  - A little
  - Moderately
  - Quite a bit
  - Extremely
3. How aware are you with efforts to combat stigma surrounding substance use disorders at your College or UTHSC?
  - Not at all aware
  - Slightly aware
  - Moderately aware
  - Very aware
  - Extremely aware
4. How satisfied are you with the efforts to combat stigma surrounding substance use disorders at your College or UTHSC?
  - Not at all satisfied
  - Slightly satisfied
  - Moderately satisfied
  - Very satisfied
  - Extremely satisfied

Participant Characteristics.

1. What College are you in?
  - Dentistry
  - Graduate Health Sciences
  - Health Professions
  - Medicine
  - Nursing
  - Pharmacy
  - Other (please specify: _______________)
2. What is your age in years? __________
3. What is the highest degree you have completed?
  - Less than high school
  - High school/GED
  - Some college
  - 4 year college degree
  - Master degree
  - Advanced health-related degree
  - Advanced non-health-related degree
4. What is the primary language spoke in your home?
  - English
  - Other (please specify: _____________)
5. During the past 3 months, how often did you worry about not being able to pay for groceries?
  - Never
  - Rarely
  - Sometimes
  - Often
  - Always
6. During the past 3 months, how difficult has it been for you and your family to afford healthcare?
  - Never
  - Rarely
  - Sometimes
  - Often
  - Always
7. During the past 3 months, how difficult has it been for you and your family to afford housing?
  - Never
  - Rarely
  - Sometimes
  - Often
  - Always
8. How many adults, including yourself, are in your household? _________
9. How many children are in your household? ___________
10. What is the zip code of the location where you have lived the longest? __________
11. To which gender do you most identify?
  - Male
  - Female
  - Transgender Female
  - Transgender Male
  - Prefer not to answer
12. How important is spirituality in your life? (rating scale)
13. How spiritual would you rate yourself?
  - Very
  - Fairly
  - Not too much
  - Not at all
14. Are you of Hispanic, Latino, or Spanish origin?
  - Yes
  - No
15. What is your race?
  - American Indian or Alaska Native
  - Asian Indian
  - Black or African American
  - Chinese
  - Chamorro
  - Filipino
  - Japanese
  - Korean
  - Native Hawaiian
  - Other Asian
  - Other Pacific Islander
  - Samoan
  - Some other race
  - Vietnamese
  - White

Thank you page and link to new survey for entry into raffle to win an Amazon gift card.

1. Thank you for completing this survey. Your responses will remain anonymous. If you would like to be entered into the raffle to win the 1 grand prize (Amazon $250 gift card) or 1/15 other prizes (Amazon $50 gift card), please follow the link below. We will contact you if you are a gift card recipient.

## Abbreviations

SUD: Substance Use Disorder
MCRS: Medical Condition Regard Scale
IRB: Institutional Review Board
DSM-5: The Diagnostic and Statistical Manual of Mental Disorders, Fifth Edition
